# The potential impact of climate change on annual and seasonal mortality for three cities in Québec, Canada

**DOI:** 10.1186/1476-072X-7-23

**Published:** 2008-05-22

**Authors:** Bernard Doyon, Diane Bélanger, Pierre Gosselin

**Affiliations:** 1 DRBEO, Institut national de santé publique du Québec, 945 ave. Wolfe, Québec, G1V 5B3, Canada; 2 Centre de recherche, CHUQ, 2705, boul. Laurier, Québec, G1V 4G2, Canada; 3 Ouranos, 550 Sherbrooke Ouest, (19e étage), Montréal, Québec, H3A 1B9, Canada

## Abstract

**Background:**

The impact of climate change and particularly increasing temperature on mortality has been examined for three cities in the province of Québec, Canada.

**Methods:**

Generalized linear Poisson regression has been fitted to the total daily mortality for each city. Smooth parametric cubic splines of temperature and humidity have been used to do nonlinear modeling of these parameters. The model, to control for day of the week and for non-temperature seasonal factors, used a smooth function of time, including delayed effects. The model was then used to assess variation in mortality for simulated future temperatures obtained from an atmospheric General Circulation Model coupled with downscaling regression techniques. Two CO_2 _emission scenarios are considered (scenarios A2 and B2). Projections are made for future periods around year 2020 (2010–2039), 2050 (2040–2069) and 2080 (2070–2099).

**Results:**

A significant association between mortality and current temperature has been found for the three cities. Under CO_2 _emission scenarios A2 and B2, the mortality model predicts a significant increase in mortality in the summertime, and a smaller, but significant decrease in the fall season. The slight variations in projected mortality for future winter and spring seasons were found to be not statistically significant. The variations in projected annual mortality are dominated by an increase in mortality in the summer, which is not balanced by the decrease in mortality in the fall and winter seasons. The summer increase and the annual mortality range respectively from about 2% and 0.5% for the 2020 period, to 10% and 3% for the years around 2080. The difference between the mortality variations projected with the A2 or B2 scenarios was not statistically significant.

**Conclusion:**

For the three cities, the two CO_2 _emission scenarios considered led to an increase in annual mortality, which contrasts with most European countries, where the projected increase in summer mortality with respect to climate change is overbalanced by the decrease in winter mortality. This highlights the importance of place in such analyses. The method proposed here to establish these estimates is general and can also be applied to small cities, where mortality rates are relatively low (ex. two deaths/day).

## Background

It now seems well established that our planet's climate is changing and that human activity is in large part responsible for this change [1]. Climate warming means, among other things, an increase in the average ambient temperature for the majority of cities, as well as a greater probable frequency of extreme meteorological events (e.g. intense heat and cold waves, ice storms, excessive rainfall).

This situation is recognized as a concern for public health [2,2,3] – since the ambient temperature can affect state of health – as well as for other spheres of activity such as land use planning (e.g., zones at risk of heat islands, flooding, erosion), infrastructure management (e.g., wastewater and potable water treatment) and buildings (e.g., air conditioning of hospitals) [4].

In recent decades, considerable effort has therefore been put into increasing understanding of mortality-climate relationships and also into developing methods and models for estimating future temperature variations.

Knowledge about the mortality-temperature relationship for a population – and, in a more general way, the relationship between mortality and any significant meteorological parameter – is vital for public health. On the one hand, this relationship allows population alert thresholds to be indirectly established when a specific climatic event is anticipated. On the other hand, it allows the potential effect of a climate change on mortality to be estimated. Several studies have already reported a significant association between daily temperature and daily mortality for different cities around the world. Several authors have reported either a U-shaped, V-shaped or J-shaped relationship to describe the effect of temperature on mortality [5-9]. Obtaining a relationship between mortality and temperature poses, in itself, a good challenge because, on the one hand, the exact shape of the relationship is not known *a priori *and, on the other, various factors can confound the results. For example, the effect of seasons on mortality must be controlled so that this effect is not confused with the effect of daily temperatures on this mortality. The literature contains a wide range of statistical methods to determine the shape of the mortality-temperature relationship [9-13].

In parallel with the advancement of research in the field of health and its link to climate, various tools and models have been developed in climate science for estimating the variations in average temperatures at the earth's surface over horizons of approximately one hundred years and spatial scaling of about 30 kilometers (km) [14,16]. Also, the scientific community has agreed on the classification of greenhouse gas emission scenarios (e.g., scenario A2), so that projections of simulated temperatures can now be compared on the basis of different climate models [17].

However, almost 20 years ago, Kalkstein [18] suggested using the output of climate models with spatial scaling comparable to the size of a city to estimate the effect of extreme climatic events on human health, but very few publications have reported doing this.

The present study aims to fill this gap. It presents the projections relating to variations in mortality, estimated using historical data and climate simulations, for three cities located in the southeastern region of the province of Québec (Canada), namely Montréal, Québec and Saguenay.

## Results

### Mortality-temperature relationship

The generic form of the model (equations 1 to 3 in the Methods section) was used first to obtain a statistical relationship between mortality from all causes (excluding trauma) and climate for the cities of Montréal, Québec City and Saguenay.

**Figure 1:**
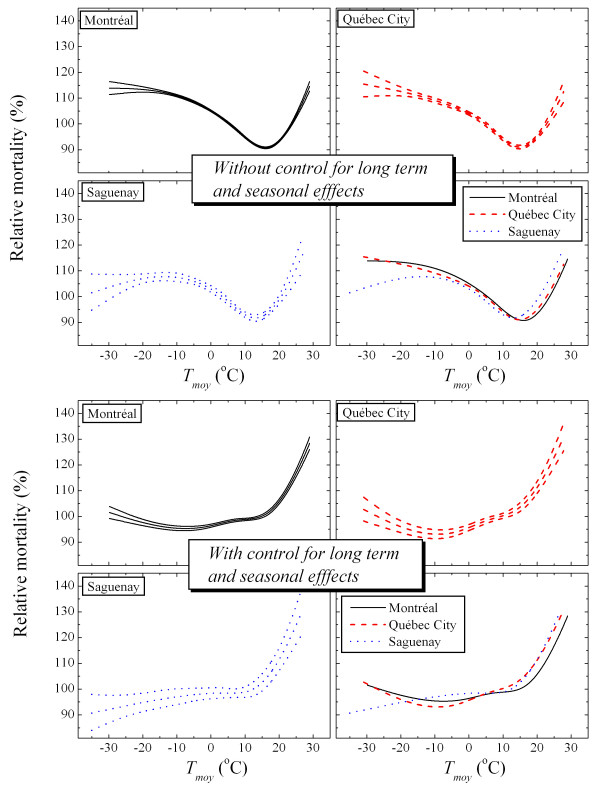
**Mortality-temperature relationship for Montréal, Québec City and Saguenay**. These graphs illustrate the relationship obtained between mortality and average daily temperature for the cities of Montréal, Québec City and Saguenay. The top graphs come from the model in which the confounding factors are disregarded (it is assumed that C = 0 in equation 2). The bottom graphs correspond to the case in which the confounding factors are taken into account (term C is added to the model with *λ*_*j *_= 7 d.f./year).

Figure 1 shows the mortality-temperature relationship for these three cities (only the relationship that dominates the effect of climate on deaths is presented in this paper). These curves represent relative mortality as a function of average temperature. For a given temperature, they allow mortality to be compared to the average of the mortalities by considering all the temperatures, while taking into account the effect of the seasons, long-term trends and days of the week (e.g., a mortality of 130% at a given temperature indicates that there are 30% more deaths at this temperature than on average). This relative mortality is directly related to the term *S*(*T*_*av*_,*4*) in equation 3. In order to highlight the importance of controlling the effect of seasons in the model, the temperature-mortality relationship without adjustment for confounding factors is also presented in Figure 1. The apparent effect of cold temperatures (the four upper graphs) is seen to be due instead to the winter season and is reduced by controlling for this factor (four graphs at the bottom of Figure 1). In addition, the slope of the linear part (temperatures above 18°C) is seen to be practically identical for the three cities. For temperatures below 18°C, no trend emerges from the graph. In fact, the effect of cold seems evident only by observing relative mortality as a function of temperatures during the weeks preceding the death, meaning by observing the results associated with the other terms in equation 3 (figures not presented here).

### Mortality estimates for simulated future temperatures

**Figure 2:**
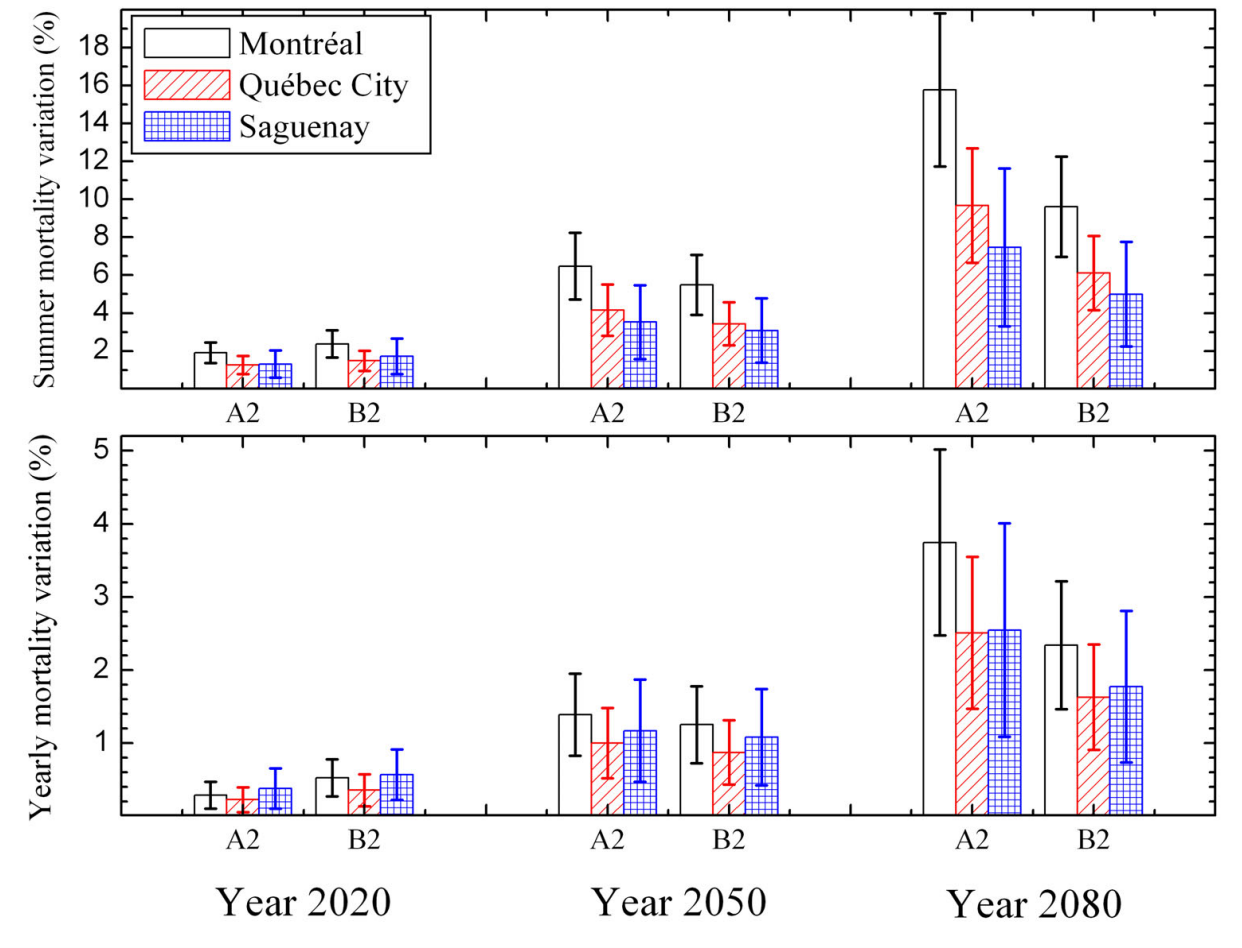
**Mortality projections under scenarios A2 and B2 for Montréal, Québec City and Saguenay**. This figure presents the variations in summer and annual mortality predicted for scenarios A2 and B2 in the cities of Montréal, Québec City and Saguenay. The variations are expressed as a percentage (%) of the historical mortality for the 1981–1999 period. The confidence interval (95%) is also given. The difference between the projections of scenarios A2 and B2 is seen to be not significant.

Using the models developed at a city scale, the variation in mortality due to climate changes could be estimated for different future periods by using future temperature projections for these periods (scenarios A2 and B2). Figure 2 shows the projected variations in summer mortality and annual mortality for the cities of Montréal, Québec City and Saguenay. The variations in mortality are expressed as a percentage of the historical mortality (1981–1999 period) and are presented for the periods around the years 2020, 2050 and 2080. The 95% confidence interval (CI) is indicated by the vertical bars. Based on the mortality projections obtained from the model, an increase of around 2% is noted for 2020 in the average summer mortality for the different cities. For 2080, this increase in summer mortality fluctuates between 8 and 15%, depending on the city. Only the predictions for the summer season were illustrated, due to the size of their variations. Annual variations in mortality have also been presented in order to cover a complete year. Although there are differences between the projections for the cities, they were not statistically significant based on a 95% confidence interval.

**Figure 3:**
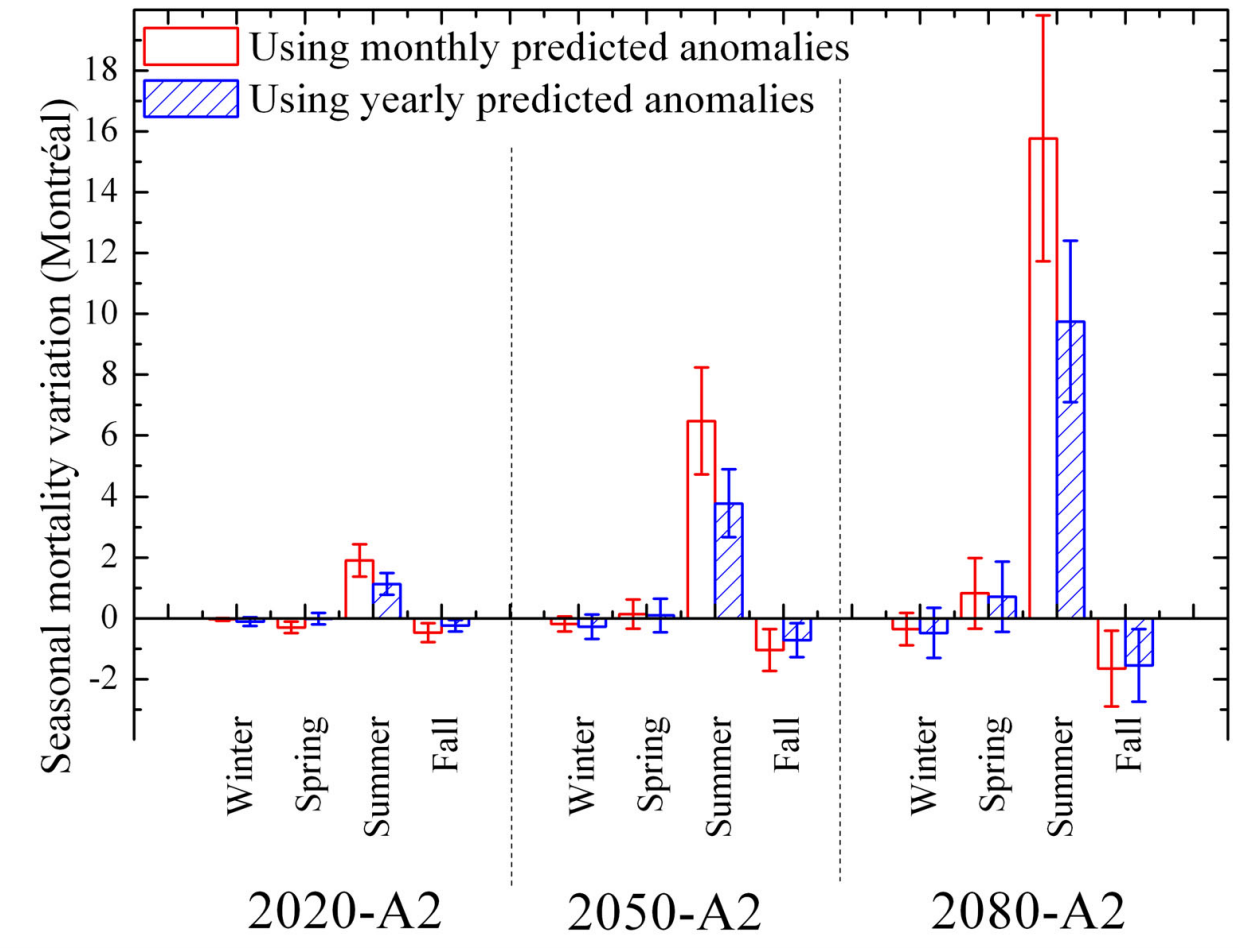
**Seasonal mortality projections under scenario A2 for Montréal using monthly and yearly future simulated temperature anomalies**. Seasonal mortality variations are presented for the city of Montréal. These variations are estimated using future temperature data obtained by adding monthly or yearly simulated temperature anomalies (A2 scenario) to historical data. Monthly or yearly temperature anomalies lead to different variations since the mortality-temperature relationship is nonlinear. Mortality variations are expressed as a fraction of the average historical mortality from 1981 to 1999. Confidence bands (95%) are illustrated with the vertical lines.

Figure 3 represents the variations in mortality by season, taking into account future scenario A2 only for the city of Montréal. In general, there is a slight decrease in mortality in winter and fall, and a slight increase in the spring. The variations in mortality during the summer are well above those observed during the other seasons, which explains the increase in annual mortality in Figure 2. In Québec City and Saguenay, the variations in mortality behave qualitatively in an identical way.

Figure 3 also illustrates the variations in mortality estimated by using, as a sequence of future data, the *annual *average anomaly added to the historical temperatures. An interesting fact is noted for the summer period, namely that the predictions obtained by adding the annual anomaly to the historical temperatures are below those resulting from the addition of the monthly anomaly. This situation reflects the existing nonlinear relationship between mortality and temperature. The contribution of a climate model that takes into account the monthly temperature anomaly should therefore be considered in establishing such estimates.

## Discussion

The literature contains a few publications presenting variations in mortality for future climate. For example, Donaldson *et al *[19] have published predictions for the United Kingdom and several of its major cities. On an annual basis, these authors predict a reduction in mortality for the periods 2020 to 2080. For each of the cities analyzed in the present study, an increase in total mortality was observed. Several arguments can explain this difference, including the capacity to adapt to cold and some methodological aspects.

As reported by Wilkinson *et al *[20], the excess winter mortality in England between 1986 and 1996 was attributed, among other things, to the lack of central heating and the high cost of heating. The eventual rise in temperature could therefore contribute to a reduction in mortality in winter in this European region. However, the situation is different in Québec. In fact, it is rather unlikely that warming will have a great impact, on average, on winter mortality. Over the years, Quebecers have developed various strategies to acclimatize to the cold. The *Act respecting the conservation of energy in buildings *(1983), aiming to ensure a minimum performance of the thermal insulation of walls and ceilings, is one example [21]. Furthermore, natural resources ensure heating at a relatively low cost, and even among the most advantageous, compared to several industrialized countries including the United Kingdom [22].

Some methodological differences between this study and that of Donaldson *et al *(2001) should be considered. First, these authors have not controlled the seasonal effect. As has been demonstrated in this paper, the control of seasons mainly affects the results for the cold part of the model (see Figure 1). A model not adjusted for this effect will predict fewer deaths for winters in future climates. By controlling the seasonal effect, an increase in mortality in summer occurs that is not balanced by a reduction for the other seasons. Adjustment for this effect is therefore essential if mortality due to climate is not to be confused with that due to seasonal factors (e.g., winter flu epidemics). Other methodological aspects differentiate this publication from that of Donaldson *et al *(2001), namely the use (in the present study) of the monthly rather than annual anomaly to construct future temperatures, almost doubling the predictions relating to variations in mortality in summer (reference Figure 3).

Using a different approach in which the concept of synoptic scale air mass is introduced to establish the relationship between mortality and climate, other researchers have estimated for several American cities that the number of deaths due to hot days would be approximately three times greater than their reduction for cold days [23]. This estimate follows along the same lines as the present study.

More recently, researchers have proposed projections for heat-related mortality for future climates in New York City [24]. Methodologically, these researchers used statistical methods similar to those presented in this publication. Nevertheless, it is difficult to compare their results directly with these because they estimated variations in mortality for future periods only for so-called hot days (meaning days above an average threshold temperature), while this present study also considers "normal" summer days and colder days (Figure 2).

Cheng *et al *[25] have made predictions for a few cities in south-central Canada, by including warming and the effects of pollution. For Montréal, 240 additional deaths related to higher temperatures are proposed for the 2080 horizon. For Montréal, Figure 2 shows an increase in summer mortality of approximately 13% (95% CI: 10%–16%) for 2080 (by taking the average of scenarios A2 and B2), which corresponds for this city to 425 additional deaths per summer (95% CI: 310 deaths-540 deaths).

It should be mentioned that the projections presented in Figures 2 and 3 were evaluated for populations that include all age groups. Analyses were done (not presented here) and show that the projected variations in mortality are greater for the age group of individuals 65 years of age and older than for the overall population [26]. With the aging of the Québec population, there will proportionally be more and more people age 65 and older (9.7% in 1986, 12% in 1996 [27] and a proportion of approximately 28% is projected for this age group in 2040 [28]). These projections can therefore be considered as a lower limit, and that with time, they will increasingly approach those obtained for a population aged 65 years and older.

Another important aspect to be considered concerns the stability of the model over time. It is difficult to predict how the population will adapt to climate changes. Some standards may possibly be implemented, mainly for the air-conditioning of dwellings. Awareness campaigns or other types of preventive interventions (e.g. urban greening, insulation) could also change the vulnerability of the people most at risk. It would therefore be interesting to redo some of these analyses using more recent data in order to compare the population's vulnerability in the 2010s to that in the 1980s.

## Conclusion

In this article, statistical models quantifying the relationship between mortality and climate for the Québec (Canada) cities of Montréal, Québec City and Saguenay have been presented. These models have been paired with local climate predictions in order to estimate variations in future mortality due to climate change. It has been estimated that the increase in summer mortality would not be balanced by a reduction in winter mortality. Thus, an increase in future annual mortality can be expected, attributable in part to the increase in average temperatures. The models proposed for establishing these estimates can be applied to cities or regions where the daily mortality is relatively low (2–3 deaths/day). The results presented in this paper concerning mortality projections are rather conservative since they do not take into account the demographic evolution of populations at risk (> 65 years of age), a person's eventual state of health, as well as possible changes in the variability of future temperatures and in the number of extreme events. The use of regional climate models could also be interesting in improving the precision of these results.

## Methods

**Figure 4:**
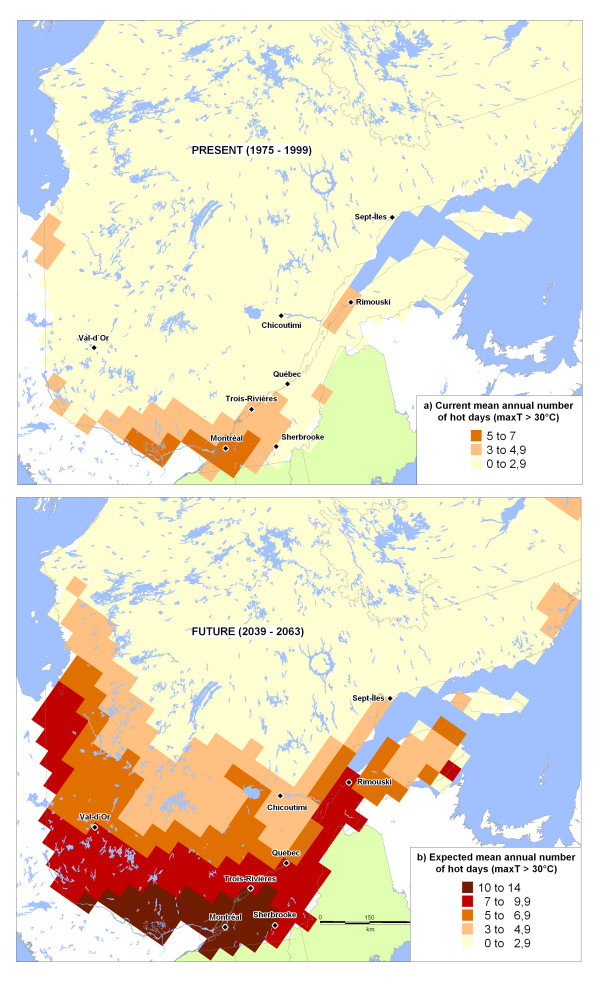
Québec with a few geographic references.

Mortality sequences (1981–1999) were studied in parallel with chronological series of several meteorological parameters (temperature, diurnal difference, humidex rating, etc.) in order to build statistical models for mortality as a function of weather. These models were then paired with the regional projections of the climate variables generated by the Ouranos Consortium [29] in order to estimate – for three cities in Québec (Montréal, Québec City and Saguenay) – the variations in average mortality for different future periods. The location of these three cities is indicated on the map of Québec presented in Figure 4.

### Studied territory and population

Québec, one of Canada's ten provinces, is located between 45 and 62 degrees (°) north latitude and between 57° and 79° west longitude. Its territory, covering an area of 1,667,441 km^2 ^[30], is bounded to the north by the Arctic Ocean, to the south by the United States and New Brunswick, to the west by James Bay, Hudson Bay and then Ontario, and to the east by the Atlantic Ocean and Labrador.

Québec has a wide range of climates with four distinct seasons. Depending on the latitude, average temperatures vary between 5 and 20°C in summer and from -25 to -10°C in winter. The total annual precipitation (rain and snow) fluctuates with the regions of the province and reaches between 500 and 1,200 mm annually.

More than 99% of the Québec population (n = 7,237,479 inhabitants) lives south of the 50^th ^parallel [30] and more than 80%, along the banks of the St. Lawrence River or its tributaries. The three cities considered in this study account for one-third of Quebecers, namely: Montréal (n = 1,812,723 inhabitants in 2001), or approximately 25% of the total population; Québec City, around 7% (n = 519,950 inhabitants in 2001); and Saguenay, approximately 2% (n = 143,692 inhabitants in 2006), and are located in a humid continental temperate climate zone [31]. Saguenay is about 450 km north of Montréal, with Québec City in between.

### Historical data

Historical data of deaths and meteorological conditions covering the 1981–1999 period for the cities of Montréal, Québec City and Saguenay were linked in a database.

Death data were obtained from the Québec Ministère de la Santé et des Services Sociaux (Ministry of health and social services). They included different information such as the date of death, the age of the deceased person, the residence postal code (truncated to 3 positions to respect confidentiality) as well as the cause of the non-traumatic deaths (codes between 1 and 799 according to the International Classification of Diseases ICD-9 [32]). For the city of Saguenay, the daily mortality is relatively low (2 deaths/day on average). This low mortality rate therefore made it possible to explore the limitations of the statistical methods used to quantify the mortality-temperature relationship.

The meteorological data from the airport stations located near the three cities studied came from Environment Canada [33].

### Simulated temperature data

The Ouranos Consortium simulated the climate data for future periods. The data were the daily maximum, minimum and average temperatures at the 2020, 2050 and 2080 horizons. These data were generated by a method known by the acronym SDSM (*Statistical DownScaling Model*), allowing the results of general circulation models (hereafter GCM) to be transposed to the local scale.

GCMs, with a spatial resolution in the order of 300 km, allow the effect of the future increase in greenhouse gases (GHGs) to be simulated on the basis of different scenarios. These GHG emission scenarios, established by the Intergovernmental Panel on Climate Change (IPCC), correspond to different evolutions in the global consumption of fossil fuels in relation to demographic, economic, and technological growth, etc. In this study, some A2 scenarios, considered as "pessimistic," and B2 scenarios, qualified as "optimistic," were considered in order to take into account the uncertainty of future GHG emissions [17] (compared to B2 scenarios, A2s suggest a greater increase in GHGs starting in the mid-21^st ^century).

The large scale synthetic data were generated by a specific GCM, known by the acronym HadCM3 [34], both for the current period (1981–1999) and for the future 2020 (i.e., 2010–2039), 2050 (i.e., 2040–2069) and 2080 periods (i.e., 2070–2099), for scenarios A2 and B2 for GHG emissions. The HadCM3 general circulation model was chosen because it generates changes in temperature close to the average of the other models, except possibly for the winter season when it is below the average [35].

These synthetic data were then brought to local scales using the SDSM method. This method, developed by Wilby and Dawson [14], is based on a representation of the statistical associations between the large scale atmospheric variables (as simulated by a GCM) and the local variables (i.e., temperatures and precipitation) using a multilinear regression. The method has been the subject of a detailed and systematic evaluation relating to its capacity to appropriately simulate climate variables and to be used to construct scenarios of climate changes in Québec [36]. A monthly average of the temperature anomaly for the different future periods was calculated by comparing historical and future synthetic data for the three cities. Other authors have also considered the monthly temperature anomaly in order to estimate mortality projections for future periods [24].

### A statistical model for mortality

Climate effects on mortality are determined mainly from methods developed for studying the health impact of air pollution [10]. The average daily mortality as a function of time and climate is estimated using a generalized linear model [37]:

(1) log *μ *= *C *+ *W*

Variable *μ *represents the *daily *expected of the number of deaths for a certain territory, a specific age group and a particular group of causes of death. The term *C *represents the confounding factors, and *W *groups the climate variables (or functions of these variables) that affect daily mortality.

Confounding factors are parameters that can affect the number of deaths without being directly associated with the daily variability in the climate. The number of deaths can vary with time. For example, more deaths occur in winter due to larger epidemics during this season [38]. Also, for a given season, an increase (or a decrease) is noted in the number of deaths attributable to the change in the size of the population if the observation period is sufficiently long [10]. Clearly, the statistical model must include a term that depends on the time and that reproduces the seasonal quasi-periodicity of the number of deaths due to these fluctuations related to seasonal cycle or long-term trends, rather than the daily variability in the climate.

The explicit form of term *C *is:

(2) *C *= *DOW *+ *S*(*j*, λ_*j*_)

Equation 2 has a different ordinate term for the days of the week (*DOW*). The term *S(j,λ*_*j*_) is a natural cubic spline parametric function of time (measured in days) with *λ*_*j *_degrees of freedom (hereafter *d.f.*), a function that reproduces the seasonal quasi-periodicity of the number of deaths and the long-term trends.

There are other possible options for controlling the seasonal effect and long-term trends. Some authors have already used month [39] or season indicators [40]; others have preferred using trigonometric functions [41-43], or even non-parametric smoothing functions [7,8,44]. In this study, the natural cubic spline parametric function was preferred over these options for the following reasons: 1/month or season indicators introduce discontinuities in time and limit the length of the time filter to two values (1 or 3 months); 2/trigonometric functions impose a periodic seasonal cycle which, in our opinion, is less appropriate because the onset and duration of epidemics can vary from one year to the next; 3/the parametric spline function was equivalent to the non-parametric smoothing functions, provided that the algorithms generating them appropriately converge [45].

The cubic spline function (equation 2: *S(j,λ*_*j*_)) is in fact a piece-wise cubic polynomial function. Each piece covers a given period, from 1.5 to 3 months, depending on the value of *λ*_*j*_. All the results presented in this paper were obtained with a value of *λ*_*j *_corresponding to 7 degrees of freedom per year, a value typically chosen in the literature [6,7,46,47]. Other values have also been considered in the analyses (from 4 to 10 degrees of freedom per year). While the shape of the mortality-temperature relationship changed slightly with these other values, the projections obtained for the variations in future mortality were not affected in a statistically significant way.

The term *W *in equation 1 groups all of the climate variables or functions of variables (maximum, minimum and average temperature, diurnal difference, humidex rating, dew point, relative humidity, atmospheric pressure and precipitation). Two important aspects must be considered to specify the form of this term. The first aspect is linked to the fact that the relationship between mortality and meteorological parameters is not necessarily linear. It is well known, for example, that cold and hot temperatures are associated with an increase in the number of deaths [5]. In order to take into account the potential nonlinear relationship between mortality and meteorological parameters, natural cubic splines functions of these different parameters were therefore introduced to construct the term *W *in equation 1. This approach is commonly used for this type of modeling [6,48,49].

The other important element to be considered in constructing the model is that past climate can affect present deaths. Therefore, the "delayed" values of the meteorological variables must be included in the term *W*. For example, for deaths on day "*j*", the temperatures *T*_*j-n *_must be introduced, namely the temperatures *n *days before the death [9]. From a statistical standpoint, this introduces a significant challenge because the estimate of the coefficients obtained by regression may be inappropriate due to the strong correlation between the temperatures. In order to avoid this problem, time groupings of the meteorological variables have been added to the model, such as the temperature of the day at the time of death, the average of the temperatures on each of the two days preceding the death, the average of the temperatures on the third day to the sixth day before the death, etc., in order to estimate the average of the deaths on day *j*. This approach, used by other authors [7,9], is sufficient to take into account the effect of past climate on mortality.

Models have been constructed by considering different combinations of maximum, minimum, and average temperatures and the humidex rating (*T*_*max*_, *T*_*min*_, *T*_*av *_and *Hx *respectively). These models have allowed only *T*_*av *_to be retained as the mortality-related temperature indicator. This variable stood out from the others statistically, based on the Akaike information criterion [50]. Different temporal groupings of the meteorological variables were then considered step-by-step.

Finally, adding a discontinuity to *W *was considered relevant. In fact, during an intense heat or cold wave, the number of deaths could be considered as increasing too rapidly to be described by a continuous cubic spline function. Two threshold terms were therefore included in the models (two binary variables: the first for the hot threshold; the second, for the cold threshold).

All of these preliminary analyses led to this generic form for the effect of climate on deaths:

(3) $$\begin{array}{c} W=S(T_{av},4)\;+\;S(T_{av1-3},4)\\ +S(T_{av4-8},4)\;+\;S(T_{av9-14},4)\\ +S(Hu_{1-3},4)+Thresh_{Hot} \end{array}$$

The first four terms are natural cubic splines functions related to the average temperature and its groupings (*T*_*av*1–3_: 1 to 3 days before the death, *T*_*av*4–8_: 4 to 8 days before the death, and *T*_*av*9–14_: 9 to 14 days before the death). The next to last term is a natural cubic spline of the average humidity from the first to the third day preceding the death. Four degrees of freedom were chosen for the cubic splines functions in equation 3. As observed during the preliminary analyses, this parameter has had little effect on the mortality projections obtained. The last term is the binary variable referring to the threshold (only the hot threshold seemed significant) defined according to this criterion – both statistical (Akaike) and practical: for a given city, the maximum *and *the minimum temperature over three days must be greater than the 98^th ^centile of their historical values.

Of all of these variables, only the term *S*(*T*_*av*_,*4*) seems statistically significant for each of the three cities (Montréal, Québec City and Saguenay). The other terms (e.g., relative humidity) can be significant or not, depending on the case. Also, in the same city, a change in parameter *λ*_*j *_(seasonal control) can change the apparent importance of a term. Equation 3 for a given city was therefore studied in two steps: 1/study of its general form; 2/exclusion of non-significant terms. As previously reported, the Akaike criterion was used to orient the choice of the model to be retained.

### Mortality projections using the model

Using the model described in the previous section, predictions were made about the variation in the number of deaths for a future period. The attempt here is to evaluate variation *due to changes in climate*. To make this prediction, it is assumed that the average of term *C *in the model (equation 1) is constant. The average relative variation in the number of deaths per day (denoted by $\langle \Delta \hat{\mu }\rangle$) can be estimated from the following calculation:

(4) $$\langle \Delta \hat{\mu }\rangle \;=\;100\;\times \frac{\langle \exp({\hat{W}}^{f})-\text{ exp(}{\hat{W}}^{h}\text{)}\rangle }{\langle \text{exp(}{\hat{W}}^{h}\text{)}\rangle }$$

This variation is expressed as a percentage (%). The averages can be taken over a year, a season or a month. Variables ${\hat{W}}^{f}$ and ${\hat{W}}^{f}$ refer respectively to the estimate of the value of term *W *in equation 1 for days in the future climate (*f*) and the historical climate (*h*). The symbol "^" is a reminder that these values are estimated from the model. Since the difference between ${\hat{W}}^{f}$ and ${\hat{W}}^{f}$ is small in average [26], equation 4 can be approximate to:

(5) $$\langle \Delta \hat{\mu }\rangle \;\approx \;100\;\times \langle {\hat{W}}^{f}-{\hat{W}}^{h}\rangle$$

To establish the average of the historical model of deaths, the historical data *observed *over 19 years (1981–1999) were used. These historical data, added to the *monthly anomaly *of the temperatures predicted by the climate models, were also used to calculate the average of the future model. The monthly anomaly for a given future period was calculated by comparing the temperatures simulated for the 1981–1999 period to the temperatures simulated for this future period.

This approach is a good starting point for establishing an order of magnitude in the predictions relating to the variation in the number of deaths that occurred locally due to global warming. On the one hand, the data obtained by downscaling are already more consistent with microclimates than the global simulations. On the other hand, the addition of monthly anomalies to the historical temperatures takes the non-linear effects of the mortality-temperature relationship more into consideration than the addition of an average annual anomaly.

## Competing interests

The authors declare that they have no competing interests.

## Authors' contributions

BD participated in the design of the study, compiled the data, performed the statistical analysis and drafted the manuscript. DB and PG initiated and participated in the design of the study and provided comments on the manuscript. All authors read and approved the final manuscript.
